# Assessing the ability of swab data to determine the true burden of infection for the amphibian pathogen Batrachochytrium dendrobatidis

**DOI:** 10.1007/s10393-016-1114-z

**Published:** 2016-04-08

**Authors:** Frances Clare, Olivia Daniel, Trent Garner, Matthew Fisher

**Affiliations:** 1 The Institute of Zoology, The Zoological Society of London, Regent’s Park, London, NW1 4RY UK; 2 Department of Life Sciences, Imperial College London, Silwood Park Campus, London, SL5 9PU UK; 3 Department of Infectious Disease Epidemiology, Imperial College London, London, W2 1PG UK

**Keywords:** emerging infectious diseases, *Batrachochytrium dendrobatidis*, infection threshold, qPCR, chytridiomycosis, *Alytes obstetricans*

## Abstract

*Batrachochytrium dendrobatidis* (*Bd*) is a pathogenic fungus which causes the disease chytridiomycosis in amphibians by infecting the animals’ epidermis. The most commonly applied method for the detection of *Bd* is the use of a sterile swab, rubbed over the keratinized areas of an amphibian and then processed to yield DNA for detection by qPCR. This method has been used to infer a threshold of lethal infection in some species; however, how reliable and reproducible the swabbing method is at detecting the true burden of infection suffered by individuals is not known. European midwife toads, *Alytes obstetricans*, are susceptible to chytridiomycosis and are highly parasitised by *Bd* across Europe. By quantifying *Bd*-load throughout the entire skin and comparing this to swab results taken from the same individual, we determined whether epidermal swabs provide a quantifiable and accurate indication of the true fungal burden suffered. Further, we examined whether we could infer a threshold for lethal infection based on comparison of swab data taken from infected *A. obstetricans* exhibiting different clinical states. From swab data, we detected significantly higher fungal burdens from moribund metamorphs compared to visually healthy individuals; however, the ability of these swab data to provide an accurate indication of the true fungal burden was not reliable. These data suggest that fungal load dynamics play an important role in disease-induced mortality in *A. obstetricans* at these sites, but that using swab data to infer an exact threshold for *Bd*-associated mortality might be inappropriate and misleading.

## Introduction

Our ability to detect emerging infectious diseases (EIDs) is critical to understanding their effects upon wildlife species and biodiversity (Mörner et al. 2002), and it is argued that rapid detection of EIDs in wildlife is essential to their management and mitigation (Voyles et al. 2014). Specific DNA-based diagnostic tests are now well established, and polymerase chain reaction (PCR)-based molecular tests have become a valuable tool in disease diagnostics (Belák et al. 2009). As a result of rapid developments in the field of molecular biology, the cost and flexibility of such methods have made them widely used in many systems. However, such approaches are not without their disadvantages; even with the advent of techniques such as real-time quantitative polymerase chain reaction (qPCR), which is widely used in disease monitoring and surveillance (Oleksiewicz et al. 2001; Picco et al. 2007; Raviv and Kleven 2009), there can still be problems with sensitivity (the method’s probability of detecting a positive individual) and/or specificity (the method’s probability of detecting a negative individual). The specificity and sensitivity of such tests may not only be important in terms of establishing an individual’s infection status (i.e. whether it is infected or not), but also in terms of estimating the number of infectious bodies with which a host is infected. In any epidemiological study, it is advantageous to be able to detect both infection status and burden simultaneously. With many pathogens, in which infectious bodies are too small to be seen by the human eye, it is important that diagnostic tests are accurate in establishing the quantity of infectious bodies within a given sample, as doing so may be key in determining the presence of dose-dependent responses and infection thresholds.

*Batrachochytrium dendrobatidis* (*Bd*) is a pathogenic fungus which causes the disease chytridiomycosis and is responsible for some of the recent global declines and extinctions of amphibian species (Stuart et al. 2004; Skerratt et al. 2007; Rosa et al. 2013). *Bd*’s ability to establish, cause disease and spread varies greatly between species and populations (Blaustein et al. 2005; Tobler and Schmidt 2010; Baláž et al. 2013). When an amphibian host is exposed to *Bd*, the outcome can range from individual resistance right through to mortality due to chytridiomycosis, which in some cases leads to severe population declines and local extinctions (Bosch et al. 2001; Retallick et al. 2004; Woodhams et al. 2007). When sampling a population for the detection of *Bd*, the most commonly used method is the use of a sterile swab which is rubbed over the skin (post metamorphosis) or the mouthparts (larval) of a subset of amphibians in the population, and then processed via qPCR, utilising a highly specific Taqman probe (described in detail by Boyle et al. 2004). qPCR has been shown to be the most rapid, specific and sensitive method available for detection of this pathogen and is the best method currently available for allowing the pathogen load to be assessed, even at very low (single zoospore) levels (Kriger and Hero 2007).

The ability of qPCR to detect an infection burden has been important in providing a mechanism by which fungal load dynamics can be assessed; Vredenburg et al. (2010) noted declines in populations of Mountain yellow-legged frogs (*Rana muscosa*) only when an average infection burden of 10,000 fungal zoospore equivalents per swab was reached, resulting in what is now widely known as ‘Vredenburg’s 10,000 zoospore rule’. These findings were echoed in a study of Crawfish frogs (*Lithobates areolatus*) (Kinney et al. 2011). Until now, it has been unclear as to how reliable and consistent the swabbing method is at detecting the true burden of infection suffered by an individual. An epidermal swab is thought to detect *Bd* DNA present on the surface of the skin; considering the life cycle of *Bd*, swabbing will not detect the reproductive life stages, which are embedded in the deeper layers of the epidermis (the stratum corneum). Maturation can take several days to complete before motile zoospores emerge onto the amphibian’s skin (Berger et al. 1998), therefore zoospores may not emerge continuously and so will not be picked up by a swab. If we were able to measure the total *Bd* infection burden an individual was suffering, we could assess how reliable swab data are at providing an accurate indication of that burden and determine whether swab data could be used to calculate an absolute indication of infection burden.

*Bd* has been detected in the highly susceptible *Alytes obstetricans* (the midwife toad) in the Pyrenees National Park, France, since 2004. Using animals from these sites, we aimed to determine whether the fungal load obtained by an epidermal swab could act as an accurate and quantifiable measure of an individual’s total fungal load. We used a technique to quantify the fungal load of the whole skin taken from *Bd*-infected *A.obstetricans*, and to compare the results to swab data collected from the same individual. Further, we assessed swab data from individuals exhibiting different clinical states, to assess whether a higher *Bd* swab load was associated with those close to death versus those who appeared visually healthy and to determine whether a threshold associated with morbidity could be assigned.

## Materials and Methods

Sampling was conducted at four *Bd*-infected lakes within the same region of the Pyrenees National Park during the summer of 2011 (Table 1). Individual *Alytes obstetricans* recent metamorphs were sampled in two physical states, visually healthy and moribund. Recent metamorphs are those which have recently emerged from the water, with a fully resorbed tail (Gosner stage 46; Gosner 1960). We considered an individual to be moribund if it lacked a righting reflex (i.e. it was unable to turn over when placed on its back), as this is a known symptom of chytridiomycosis (Berger et al. 2005b). Visually healthy individuals possessed a righting reflex and were strong and alert with no signs of skin damage. Sterile cotton swabs (MWE medical wire) were gently rotated over the hind legs, feet and pelvic patch (five swipes/turns on each area) of each recent metamorph. All swabs were stored in dry tubes at 4°C until processing could take place. Where possible, a sample size of 30 individuals per state was collected. Any moribund individuals were euthanised using an overdose of MS222 (tricaine methanesulfonate, 1000 mg/L, buffered to neutral pH using bicarbonate of soda) (Torreilles et al. 2009), and subsequently stored in 70% ethanol. For ethical reasons, no visually healthy animals were euthanised, and the skin digest technique was performed on moribund individuals only.

**Table 1:** Summary Information for Each of the Four Lakes Sampled.

Lake	Longitude	Latitude	Area (ha)	Altitude (m)	*Bd* detected
Ansabere	0°42′30.64″W	42°53′13.91″N	0.2	1859	2005
Arlet	0°36′54.12″W	42°50′24.20″N	2.7	1986	2005
Lhurs	0°42′14.30″W	42°55′17.84″N	1.5	1691	2009
Puits	0°38′0.20″W	42°51′51.05″N	0.2	1867	2006

We followed the protocol of Boyle et al. 2004, to quantify *Bd* prevalence and infection burden in swab samples, as assessed by quantitative PCR (qPCR). To avoid inhibition, all extractions were diluted 1:10 prior to qPCR; therefore, results were multiplied by 10 in order to determine the undiluted value of the infection burden (Boyle et al. 2004). Following the protocol of Hyatt et al. (2007), we added a VICTM-labelled synthetic amplicon as an internal positive control (IPC) to a subset of samples (50%), in order to test for the presence of PCR inhibitors. We defined infection intensity/burden as the number of zoospore genomic equivalents (GE) per swab. All samples were run in duplicate, and a sample was assigned a positive reading if both wells amplified and an average estimate of 0.1GE or above was produced, when comparing the sample to the standard curve generated by the standards.

**Figure 1:**
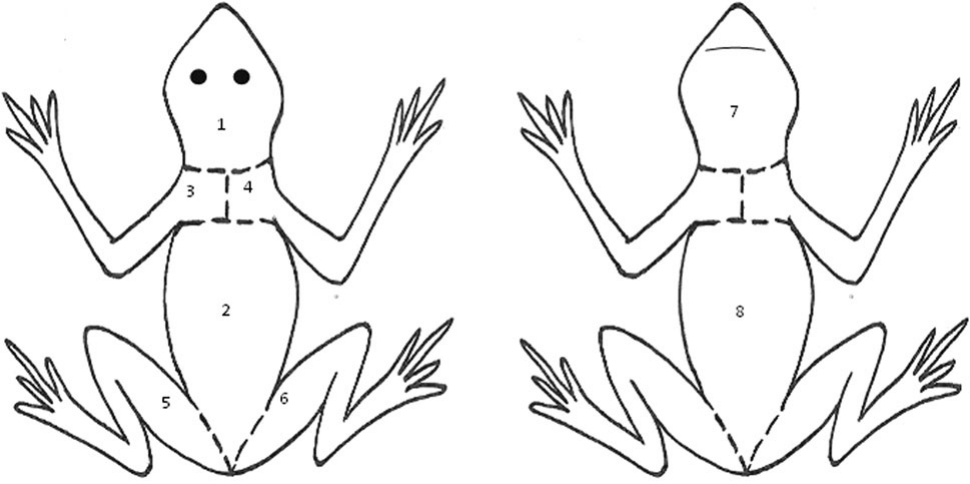
Illustration of cut locations, denoted by the *dashed line*, dividing the entire skin of an individual *A. obstetricans* metamorph into eight separate sections. Cut locations are identical on the dorsal and ventral surface, with each front and hind limb being one whole skin section.

To evaluate the quantity of *Bd* infecting the entire skin of moribund metamorphs, individuals were carefully skinned using forceps and a sterile scalpel blade. The skin was divided into eight sections so as not to overload the spin columns. Sections included (1) dorsal head; (2) dorsal back; (3) left front limb, including shoulder and upper chest; (4) right front limb, including shoulder and upper chest; (5) left hind limb; (6) right hind limb; (7) ventral head and (8) ventral abdomen (Fig. 1). We considered the sum total of all eight skin sections to equal the total burden of *Bd* infecting that individual. Each skin section was finely diced with a sterile scalpel blade prior to placement in a 1.5-ml microcentrifuge tube. Skin samples were extracted using the DNeasy Blood & Tissue Kit (Qiagen). The spin-column Qiagen protocol for use with animal tissue was closely followed with the following two modifications: (1) an extra homogenisation step following the addition of Buffer ATL, and (2) an increased incubation period (~20 h) following the addition of proteinase K. In order to increase overall DNA yield, we repeated the final step, so a total of 400ul of eluate was collected per sample. All samples were stored at −20°C prior to analysis.

Ten microlitres of elute from the digest samples was diluted following a 10–10,000 dilution series using molecular grade sterile water, in order to determine which dilution factor gave the optimal results. An IPC was spiked into a subset of samples, at all dilution levels to assess inhibition as described above. We determined that a dilution factor of 1000 provided the most reliable results, with the least inhibition, so all skin digest samples were diluted to this factor in preparation for qPCR analysis, as described above. Ten microlitres of the 400 µl elute was used to make the 1000 dilution (in 90 µl sterile water), and 5 µl of this dilution was used for each qPCR reaction. Therefore, all digest qPCR results were multiplied by 800,000 to obtain a quantitative estimate of zoospore genomic equivalents present throughout the entire skin sample from each individual.

## Statistical Analysis

All statistical analyses were carried out using the statistical package ‘R’, version 3.0.0 (R Core Team 2013). All *Bd* DNA scores (GE) were rounded to the nearest whole number and treated as count data (all samples between 0.1 and 0.9 were assigned a (1) and negative binomial regression models (function glm.nb from the R-package MASS) were used to search for any differences in the GE scores detected by a swab between the visually healthy and moribund individuals, using the combined GE from all lakes. Likelihood ratio tests were used to assess the models’ overall significance. A series of linear models were applied to determine a correlation between the amounts of *Bd* DNA detected via a swab compared to that detected via the skin digest method, for the same individuals. Comparisons were made between the swab result and the combined value of the digest results from the pelvic patch (PP) plus the hind limbs, as this is the area commonly swabbed, and also the swab compared to the entire digest for each individual.

## Results

**Figure 2:**
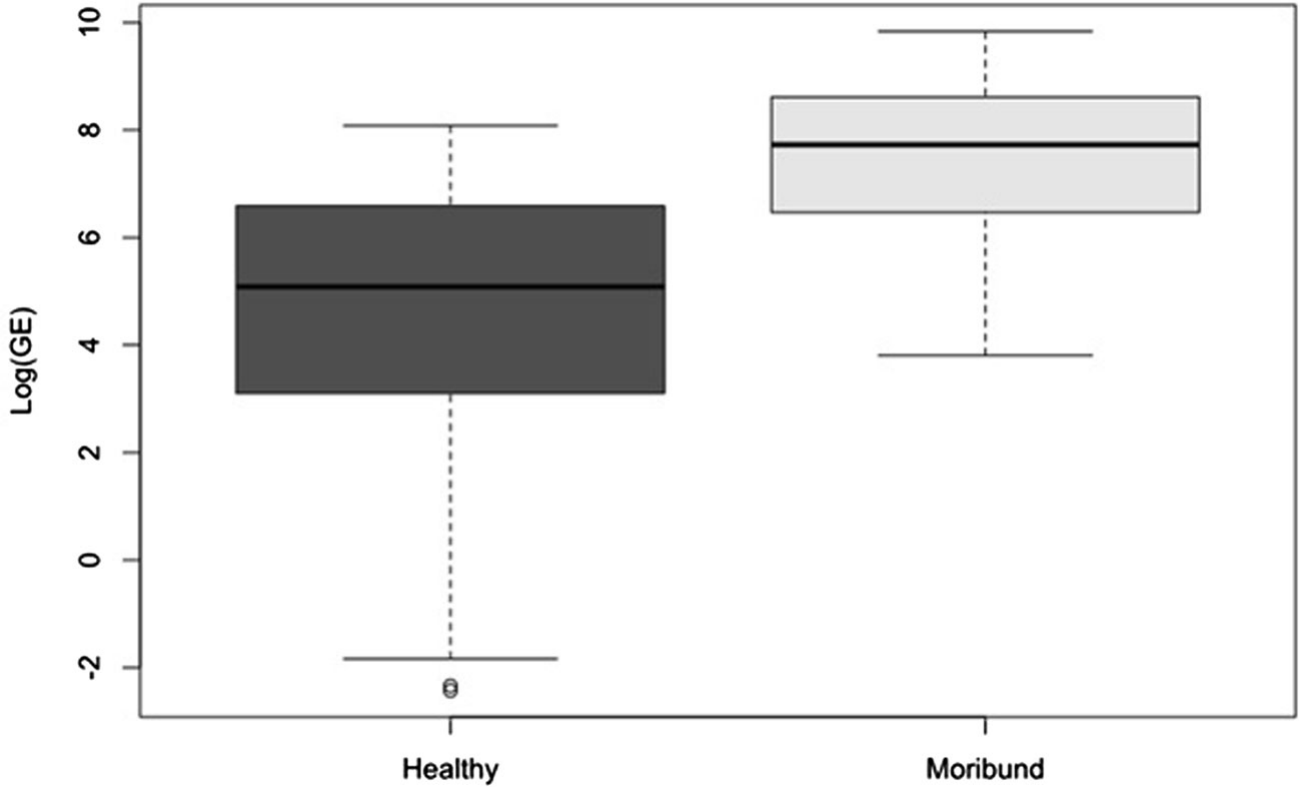
Fungal burden (Log GE) detected in swabs taken from the visually healthy metamorphs (*dark grey*, *n* = 94) and moribund metamorphs (*light grey*, *n* = 17) from all lakes combined.

Swab samples were taken from 30 visually healthy individuals from each of lac Arlet and lac Puits, 27 from lac Lhurs and eight from lac Ansabere. *Bd* was detected in all but two metamorphs across all four lakes sampled; the two negative individuals were removed from all of the following analysis. A total of 17 swabs were taken from moribund metamorphs, across all four lakes; (Arlet = 10; Lhurs = 5, Puits = 1, Ansabere = 1) (Table 2). Although some inter-lake differences in infection burden were seen in the visually healthy group, these differences were not as pronounced as those seen between the different states (i.e. healthy-moribund); therefore, due to the small sample sizes per lake in the moribund state, we combined data from all lakes to assess the differences in infection burden detected in swabs between different states. Visual state was a significant predictor of infection burden within individuals (*χ*^2^ = 37, df = 1, *P* < 0.001). The infection burden detected in the moribund group was significantly higher than that detected in visually healthy group (*z* = 5.326, df = 109, *P* < 0.001) (Fig. 2). IPC results revealed that no inhibition was present within the swab samples.

**Table 2:** Summary of Fungal Infection Burden (in Genomic Equivalents) Detected in Swabs from all Lakes Combined in Both Visually Healthy and Moribund Metamorphs.

State	Sample size	Average infection burden (±1 SE)	Infection burden range
Visually healthy	94	473 (±73)	0.1–3248
Moribund	17	4018 (±1169)	45–18,734

**Figure 3:**
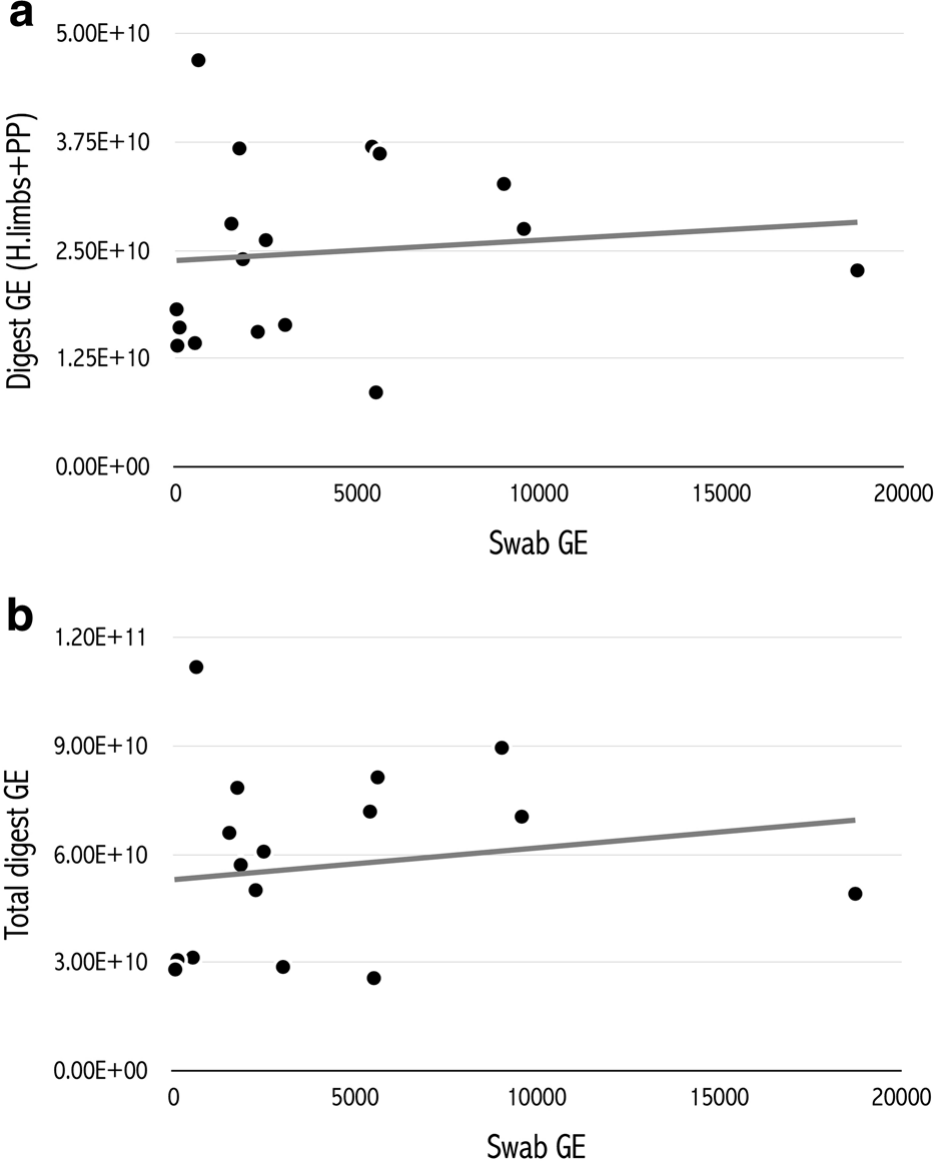
Relationship between the fungal burden detected via a swab (Swab GE) compared to **a** the combined fungal burden detected in the skin of the hind limbs and pelvic patch (digest GE HL & PP) and **b** in all skin sections combined (Total digest GE), obtained via a full-skin digest, in moribund individuals (*n* = 17).

We did not detect a significant correlation between the swab GE and the GE from the combined value of those parts of the body usually swabbed for *Bd*, the pelvic patch and hind limbs skin digest (PP + HL) (linear regression: *t* = 0.42, *P* = 0.68), or between swab GE values and the combined GE for all skin digest sections (total digest) (*t* = 0.647, *P* = 0.53) in moribund individuals (Fig. 3). *Bd* DNA was found to be relatively evenly distributed among skin sections of the body with average GE ranging from 4.93 × 10^9^ (Back) through to 9.44 × 10^9^ (right front limb), Table 3. Averaged GE totals for the whole animal were 5.66 × 10^10^, compared to 4018 for the averaged epidermal swab data.

**Table 3:** Amount of *Bd* DNA (Displayed as Genomic Equivalents (GE)) Detected in Each Skin Section of Each Individual *A. obstetricans.*

Lake sampled	Dorsal head	Ventral head	R front limb	L front limb	Pelvic patch	Back	Right hind limb	Left hind limb	Swab	Digest: PP + hind limbs	Total digest
Ansabere	1.51E + 09	2.86E + 09	1.25E + 10	8.46E + 09	8.08E + 09	9.34E + 09	8.59E + 09	9.50E + 09	2495	2.62E + 10	6.08E + 10
Arlet	6.77E + 08	7.12E + 09	1.01E + 10	4.93E + 09	5.86E + 09	3.57E + 09	7.50E + 09	9.31E + 09	18735	2.27E + 10	4.91E + 10
Arlet	3.21E + 09	1.81E + 09	5.28E + 09	5.92E + 09	4.05E + 09	9.12E + 08	2.89E + 09	1.66E + 09	5519	8.60E + 09	2.57E + 10
Arlet	5.74E + 09	7.28E + 09	8.99E + 09	7.77E + 09	5.34E + 09	4.73E + 09	5.23E + 09	4.99E + 09	2274	1.56E + 10	5.01E + 10
Arlet	1.91E + 10	9.09E + 09	1.85E + 10	1.10E + 10	1.61E + 10	7.81E + 09	1.75E + 10	1.33E + 10	644	4.70E + 10	1.12E + 11
Arlet	8.96E + 08	4.95E + 09	5.58E + 09	2.91E + 09	3.29E + 09	2.72E + 09	7.56E + 09	3.49E + 09	548	1.43E + 10	3.14E + 10
Arlet	2.39E + 09	9.08E + 09	1.47E + 10	3.86E + 09	1.00E + 10	4.92E + 09	1.05E + 10	1.64E + 10	5413	3.70E + 10	7.19E + 10
Arlet	2.18E + 09	1.41E + 10	1.63E + 10	4.15E + 09	8.69E + 09	8.44E + 09	1.42E + 10	1.33E + 10	5622	3.62E + 10	8.14E + 10
Arlet	1.58E + 10	1.27E + 09	1.26E + 10	1.28E + 09	5.14E + 09	6.96E + 09	5.19E + 09	1.77E + 10	1550	2.81E + 10	6.60E + 10
Arlet	1.74E + 10	7.67E + 09	1.19E + 10	1.46E + 10	1.26E + 10	5.33E + 09	1.19E + 10	8.16E + 09	9031	3.27E + 10	8.96E + 10
Arlet	3.80E + 09	1.04E + 10	9.77E + 09	9.34E + 09	9.06E + 09	9.70E + 09	9.84E + 09	8.63E + 09	9578	2.75E + 10	7.05E + 10
Lhurs	6.31E + 09	3.21E + 09	3.11E + 08	1.11E + 09	5.19E + 09	1.37E + 09	7.52E + 09	3.73E + 09	3025	1.64E + 10	2.88E + 10
Lhurs	6.53E + 08	1.36E + 09	7.18E + 09	1.58E + 09	6.14E + 09	5.07E + 08	5.05E + 09	7.04E + 09	45	1.82E + 10	2.95E + 10
Lhurs	6.87E + 08	4.16E + 09	5.98E + 09	1.58E + 09	3.67E + 09	2.19E + 09	7.58E + 09	4.86E + 09	127	1.61E + 10	3.07E + 10
Lhurs	1.98E + 09	3.93E + 09	2.03E + 09	2.86E + 09	8.25E + 09	3.30E + 09	4.63E + 09	1.12E + 09	66	1.40E + 10	2.81E + 10
Lhurs	2.95E + 09	9.77E + 09	6.38E + 09	6.68E + 09	1.01E + 10	7.28E + 09	9.20E + 09	4.68E + 09	1864	2.40E + 10	5.71E + 10
Puits	5.90E + 09	5.94E + 09	1.23E + 10	1.28E + 10	9.82E + 09	4.70E + 09	1.05E + 10	1.65E + 10	1770	3.68E + 10	7.85E + 10
AVERAGE	5.37E + 09	6.12E + 09	9.44E + 09	5.93E + 09	7.74E + 09	4.93E + 09	8.55E + 09	8.50E + 09	4018	2.48E + 10	5.66E + 10

The combined amount of *Bd* DNA detected in the pelvic patch and hind limbs (PP + hind limbs), the overall amount of *Bd* DNA, i.e. all skin sections combined (total digest) and the *Bd* DNA detected by swab for that individual. The overall average from each section, swab, PP + legs digest and total digest is also shown.

## Discussion

The aim of this research was to determine whether swab data could provide an accurate measure of an individual’s total/full fungal burden, as assessed using a full-skin digest. Our results suggest that the data obtained by a swab are not an accurate representation of the true burden of infection suffered by an individual, as there was no correlation seen between the two methods. Taking a swab sample from the surface of the skin may merely give an indication of the current zoospore shedding/activity rate. It is likely that the zoospore shedding rate will vary depending on what stage the lifecycle is at, and various factors have been shown to influence the rate of this cycle. *Bd* zoospores survive in water and are quickly killed by drying (Johnson and Speare 2003), so if it happened to be a particularly hot and dry day when swabbing, emergence of the zoospores may be slowed in order to avoid unfavourable conditions. While the value of swab data is still high, and currently the only reliable, non-invasive method capable of quantifying *Bd* infection load, we believe that providing absolute thresholds of infection associated with mortality or decline based on swab data could be misleading owing to the high variance that we found.

The current school of thought on the distribution of *Bd* throughout the cutaneous surfaces of metamorphic animals is that infection is concentrated on the ventral surface, especially within the pelvic patch, thighs and digits, with often little infection detected elsewhere in the body (Berger et al. 1998, 2005a; Pessier et al. 1999). Results are based on histological examination of infected skin sections taken from the dorsal and ventral surface of different species and histology is usually only performed on a very small skin section, providing a snap-shot of the infected area on the whole; until now, no quantitative studies have been carried out to further assess this distribution (Berger et al. 2005b). Interestingly, our skin digest data indicate that the distribution of *Bd* is relatively consistent throughout the body in *A. obstetricans* recent metamorphs. It has been suggested that in order to detect infection, the ideal areas to swab a metamorphic or adult amphibian are the pelvic patch and hind limbs (North and Alford 2008). Although our data indicate that the distribution of *Bd* is relatively consistent, it is possible that the areas suggested are indeed the most suitable for swabbing; the pelvic patch and hind limbs may expel the highest quantity of zoospores as these areas come into contact with moisture more frequently than the rest of the body, which is likely to stimulate *Bd* zoosporangia to shed their mature zoospores. However, although a different family of amphibian, a recent study has shown that swabbing dorsal body surfaces was more effective at detecting *Bd* when compared against swabbing the ventral surfaces of caecilians (Gower et al. 2013).

We found that infection burden detected by a swab from moribund individuals was significantly higher than that detected by a swab in the visually healthy individuals, indicating that fungal load dynamics play an important role in disease-induced mortality at these sites. However, what is evident is that there is a large overlap in individual swab values between and among states. With some confidence, and in agreement with Vredenburg (Vredenburg et al. 2010), we can infer that a high infection burden, of >1000GE, is likely to increase the risk of mortality in this system, based on swab data. However, we also show that the absence of a high burden of infection, as detected by a swab, is not necessarily an indication of a healthy state. Some individuals were close to death with a relatively low swab GE (i.e. in the hundreds), whereas some appeared visually healthy whilst exhibiting a high GE (i.e. in the thousands). Previous studies have often inferred a threshold of infection, associated with mortality and population decline, based on the average swab GE suffered by a small group of metamorphs (Vredenburg et al. 2010; Kinney et al. 2011). Considering parasite/pathogen data are usually unevenly distributed (Crawley 2007), in reality assessing a threshold of infection based on averages, especially from a small sample size could be misleading: physical state should also be considered carefully. For instance, determinations of whether amphibians are moribund may be confounded by antipredator responses, whereby animals ‘play dead’. We recently showed that elevated levels of the stress hormone corticosteroid were associated with an impaired righting reflex in *A. obstetricans*, a defined experimental endpoint for many amphibians (Gabor et al. 2015). Therefore, future investigations could also take into account other biomarkers of clinical disease, beyond simply qPCR data from epidermal swabs. Although additional causes of mortality cannot be ruled out, in the past histology has been conducted on the focal species across Iberia, including the sites investigated here in the Pyrenees (Bosch et al. 2001; Walker 2008). The cause of death has been historically determined as chytridiomycosis at these sites and the mortality patterns and signs of ill health we have seen are completely consistent with these previous studies. We therefore feel confident that chytridiomycosis is the most likely cause of mortality in the individuals examined in this study.

In conclusion, while the value of swab data is high, especially for the possibility of determining a low, medium or high burden of infection, the lack of correlation between the two methods implemented here indicates that data obtained by swabbing individuals are not necessarily representative of the true burden of infection. Because of the limited ability of swab data to assess true burden of infection, we must therefore be careful when using it to assign a threshold of infection associated with mortality.
